# Increased acid sphingomyelinase levels in saliva as oral mucositis severity predictors

**DOI:** 10.3389/fonc.2025.1613884

**Published:** 2025-09-25

**Authors:** Elsa Beatriz Monroy Ordonez, Tanja Sprave, Andreas R. Thomsen, Henning Schäfer, Anca-Ligia Grosu, Verena Jendrossek, Michael Henke, Kristian Unger, Diana Klein

**Affiliations:** ^1^ Department of Radiation Oncology, University Hospital of Freiburg, Freiburg, Germany; ^2^ German Cancer Consortium (DKTK) Partner Site Freiburg, German Cancer Research Center, Freiburg, Germany; ^3^ Faculty of Medicine, University of Freiburg, Freiburg, Germany; ^4^ Institute for Cell Biology (Cancer Research), University Hospital Essen, University of Duisburg-Essen, Essen, Germany; ^5^ Department of Radiation Oncology, University Hospital, Ludwig Maximilian University of Munich (LMU) Munich, Munich, Germany; ^6^ Research Unit Translational Metabolic Oncology, Institute for Diabetes and Cancer, Helmholtz Zentrum München Deutsches Forschungszentrum für Gesundheit und Umwelt (GmbH), Neuherberg, Germany; ^7^ Bavarian Cancer Research Center (BZKF), Munich, Germany; ^8^ German Cancer Consortium (DKTK), Partner Site, Munich, Germany

**Keywords:** head and neck cancer, radiation therapy, oral mucositis, biomarker, acid sphingomyelinase, ASM, SMPD1, saliva

## Abstract

**Introduction:**

Radiotherapy (RT) plays a central role in multidisciplinary treatment approaches in cancer therapy, particularly as an effective primary treatment modality for patients with head and neck cancer (HNC). One of the most common acute complications of RT for HNC patients is radiation-induced oral mucositis (OM), which can lead to severe oropharyngeal pain, swallowing and speech difficulties, and weight loss, thereby eventually causing interruption of RT. Although OM varies with tumor location and treatment methods, it is overall a common occurrence. However, it is unclear in which patients suffer from this severe condition. This study aimed to evaluate the suitability of acid sphingomyelinase (ASM) as a potential biomarker for predicting the risk of OM and to investigate the association with OM severity.

**Methods:**

We investigated two independent patient cohorts from consecutive prospective studies (n=187). ASM protein levels were analyzed using Western blot analysis in unstimulated saliva samples collected from respective patients at least three days before the RT started. Patients were stratified according to OM occurrence and severity. Group comparisons were performed using non-parametric tests, while logistic regression was applied to assess associations between ASM levels and early OM development. Kaplan-Meier and Cox regression analyses evaluated correlations with overall and recurrence-free survival.

**Results:**

In the first cohort, 74 out of 109 patients developed OM during RT, and 42 displayed early OM at low radiation dose. Grade 3 OM developed in 50 (67.6%) patients after definitive and 24 (32.4%) after adjuvant RT. Thirty-four patients did not develop OM. A significant increase in ASM levels was detected in the saliva of patients who developed OM early. Respective findings were confirmed in a second cohort (n=78). 44 out of 78 patients developed OM, of which 21 patients displayed early OM. Fifty-three patients did not develop OM. Elevated ASM levels were confirmed in the saliva of patients who developed OM early, an observation that was found particularly in the saliva of HPV-negative patients. HPV-positivity was present in 32 (41,0%) patients. Overall, regression-free survival did not correlate with the incidence of OM or HPV status.

**Conclusion:**

Although there is currently limited evidence for the potential implementation of salivary biomarkers to assess their association with the severity of OM, the findings here show that determining ASM levels in the saliva of HNC patients before starting RT could be a promising method to predict OM risk.

## 1 Introduction

Radiotherapy-induced oral mucositis (OM) in patients with head and neck cancer (HNC) is a frequently reported acute toxicity (1, 2). The overall OM incidence for all degrees of OM ranges between 60 and 100% (2–5). Acute OM 1–2 grades are characterized by redness, tissue destruction and pain. Further escalation of symptoms of OM to grade 3 and higher leads to severe ulcers. OM not only significantly impairs the patient’s quality of life; due to the burdensome oral discomfort and dysfunction, this secondary complication leads to increased treatment costs (6). Therefore, predictive markers that identify patients at (high) risk for OM and are also easily detectable would be of great interest. Saliva, as a very easily accessible body fluid, appears to serve as an indicator of oral disease status and is therefore predestined for the identification of sensitive and/or specific protein biomarkers. A number of potential biomarkers have already been described, mostly based on proteomic approaches (7). The presence of IL-6, IL-10, and TNF-α in the saliva of HNC patients during treatment, for example, have already been described as predictors of the occurrence and severity of OM (8). Many of these pro-inflammatory cytokines (e.g., TNF-α and IFN-γ) exhibit apoptosis-inducing properties and are known to impact to epithelial cell integrity via generation of the second messenger ceramide generation (9–11). Its role in mucositis -although mainly gastrointestinal mucositis – becomes increasingly clear (11–13). Ceramide in turn is generated through degradation of sphingomyelin by acid sphingomyelinase (ASM), a well-studied lysosomal enzyme known as intermediate signaling enzyme in cell death and inflammation processes (14). At the same time, elevated plasma or serum ASM levels were found to be increased under various pathological conditions (15). The detection of ASM in human saliva and its advantages in diagnosis has already been described for other diseases, e.g., for Niemann-Pick disease (16, 17), and salivary biomarker identification for oral cavity diseases (18, 19) and/or oral cancers (20, 21) particularly for OM prior cancer treatment could help to protect against subsequent therapy-induced OM (22, 23). Here, we examined the biomarker potential of ASM, in particular whether its presence in the unstimulated saliva of HNC patients prior to radiotherapy can be used to predict the occurrence of oral mucositis.

## 2 Material and methods

### 2.1 Patient cohort

This analysis includes two separate patient cohorts from two different consecutive prospective studies. For a better distinction and comparison of the results, we shall refer to the first patient cohort as “historic/retrospective” and the second as “prospective”. The first retrospective study included HNC patients undergoing primary definitive or adjuvant (chemo)radiotherapy (C)RT) at the University Hospital of Freiburg between 2008 and 2015. In the following, this cohort is labeled as historical. The primary objective of this study was to evaluate the value of protein profiles in blood serum and saliva for predicting severe OM and clinical OM assessment under (C)RT. All procedures were approved by the Ethics Committee of the University of Freiburg (vote ETK-FR 30/10). The other prospective study was conducted at the University Hospital of Freiburg as previously described (4) and included HNC patients treated between 2017 and 2022. The purposes of this study were to analyze the role of oral keratinocytes in predicting severe OM (4), and systematic assessment of mucositis during (C)RT. All procedures were approved by the Ethics Committee of the University of Freiburg (vote ETK-FR 449/16, amended by vote ETK-FR 413/17). Written informed consent was obtained in all patients. All personal data and biopsy samples were de-identified and anonymously analyzed. All patients were discussed in a multidisciplinary tumor conference. All HNC were confirmed by biopsy. Chest MRI and/or CT staging was performed to exclude distant metastases prior to any decision on multimodal therapy. Systemic treatment was performed according to current guidelines and tumor board recommendations. In summary, definitive CRT was recommended for locally advanced and unresectable tumors. In adjuvant cases, CRT was guided by surgical pathology findings. Patients in the prospective cohort were staged according to TNM/AJCC 8th edition and 7th edition in the historical cohort. The prescribed dose for definitive CRT was 70 Gy EQD2 to the primary tumor region, for patients undergoing adjuvant RT, 60–66 Gy EQD2 to the tumor cavity. CT-based (Brilliance, CT Big Bore, Philips, Cleveland, OH, USA) three-dimensional treatment planning was performed (Oncentra MasterPlan, Nucletron, Veenendaal, the Netherlands; and Eclipse™ planning systems, Varian Medical Systems, Palo Alto, CA, USA), using individually collimated portals (6 or 18 MV; Synergy; Elekta, Crawley, United Kingdom), IMRT or volumetric modulated arc therapy (VMAT) were used. All patients received image-guided radiotherapy and were followed every three to six months by a surgeon and a radiation oncologist for the first two years, after which annual examinations were scheduled. All local recurrences were confirmed via histology. Participants with a smoking history of at least 10 pack-years were considered as smokers.

### 2.2 Mucositis scoring

OM was regionally assed (oral cavity including lips, tongue, right and left buccal mucosa, soft palate, hard palate and floor of mouth) and reactions were scored using the National Cancer Institute — Common Toxicity Criteria (NCI-CTC v3.0/4.0) in twice weekly assessments (4, 24, 25). The exact time point and therefore irradiated total dose until the appearance of grade III mucositis was recorded and calculated for each cohort using the following classifications. Retrospective cohort: the median dosis for grade 3 mucositis is 34 Gy: (i) Patients who did not have grade 3 mucositis were grouped as r++; (ii) Patients who had grade 3 mucositis >= 34 Gy were grouped as r; (iii) Patients who had mucositis with < 34 Gy were grouped as s. Prospective cohort: the median dosis for grade 3 mucositis is 31.5 Gy: (i) Patients who did not have grade 3 mucositis: were grouped as r++; (ii) Patients who had grade 3 mucositis >= 31.5 Gy were grouped as r; (iii) Patients who had mucositis with < 31.5 Gy were grouped as s.

### 2.3 Saliva collection

Saliva was collected from patients at least three days before the radiotherapy started as previously described assessments (24). In brief, patients were asked to sit head forward and to let saliva just float out of the mouth for 10 min into a funnel placed onto a 50 mL conical tube, kept in an ice cup. Collected saliva was immediately centrifuged at 3000 rpm, for 15 min and 4°C to remove insoluble material. One mL of the supernatant was pipetted and mixed with 2 μL protease inhibitor cocktail as well as 3 μL of 1 mM sodium orthovanadate solution (both Sigma Aldrich, St. Louis, MO, USA)., aliquoted and stored at −80°C until downstream analysis.

### 2.4 Data analysis and statistics

All statistical analyses were performed using R (4.4.2) within an R Markdown workflow. Descriptive statistics were used to summarize clinical and molecular characteristics. Categorical variables were compared using Chi-square or Fisher’s exact tests, and continuous variables were analyzed using Wilcoxon rank-sum tests, as appropriate. Survival analyses were conducted using the Kaplan-Meier method, and group differences were assessed using the log-rank test. Multivariable survival analyses were performed using Cox proportional hazards regression models to evaluate the association between clinical variables and with overall survival, locoregional survival or recurrence-free survival. Model assumptions were tested using Schoenfeld residuals (cox.zph), and model discrimination was assessed using the concordance index (C-index). Hazard ratios (HRs) with 95% confidence intervals (CIs) were reported. Additional subgroup analyses were carried out by comparing each mucositis category against all other groups using stratified log-rank tests. Tables summarizing baseline characteristics and their associations with clinical outcomes were created using the tableone and gtsummary packages. All tests were two-sided, and p-values < 0.05 were considered statistically significant.

### 2.5 Western blotting

The saliva samples were thawed on ice and centrifuged (5 min, 2000 rpm) to remove cell debris. The supernatant was collected and subjected to protein analysis using thePierce™ BCA Protein Assay Kit (#A55864; ThermoFisher Scientific). After denaturation with Laemmli buffer, the samples were separated using SDS gel electrophoresis. Equal amounts (50 µg of total proteins) were used. Ponceaus S Staining Solution was used to visualize blotted proteins (#A40000279; ThermoFisher Scientific). Western blots were done as previously described (26). The goat-anti-ASMase antibody was kindly provided by Prof. K. Sandhoff (Bonn, Germany) (27). Densitometric quantifications of the Western blot signals of respective signals were quantified using ImageJ (https://imagej.net/ij/index.html).

### 2.6 ASMase activity assay

Acid Sphingomyelinase Assay Kit (#ab252889; Abcam) was used according to the manufacture’s instruction for measuring ASMase enzymatic activity using colorimetry (OD 570 nm). ASMase activity (mU/mL) was determined following substrate conversion, sphingomyelin to phosphorylcholine and ceramide at pH 5.0 and 37°C (30–60 min). Optical densities (OD) were determined and activity was calculated using a choline standard curve: Sample Acid Sphingomyelinase Activity = B/(T X V) x D = nmol/min./ml = mU/ml (B = choline amount from the standard curve (nmol) T = time (mins) V = sample volume added into the reaction well (mL) D = sample dilution factor).

## 3 Results

**Figure 1:**
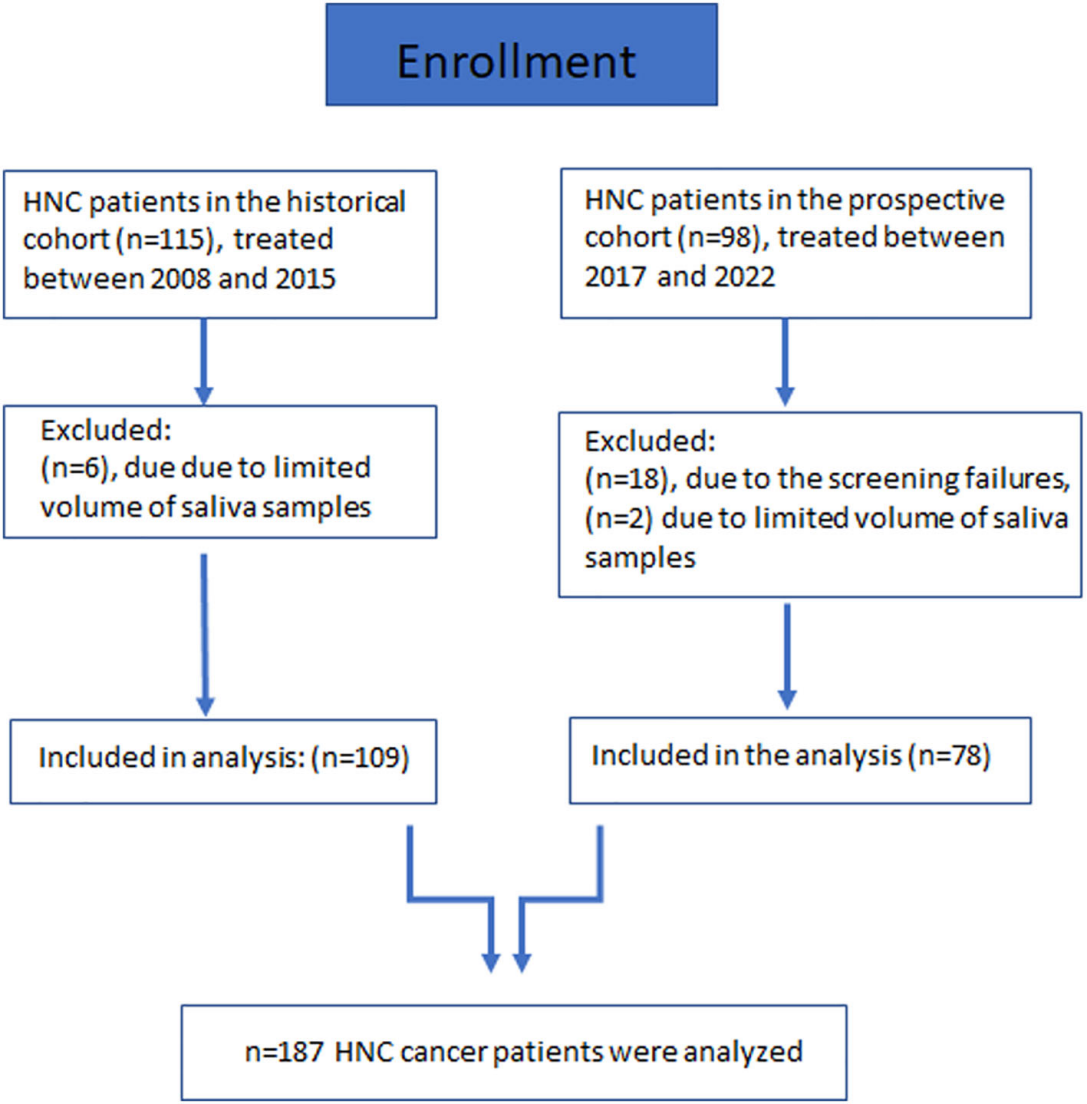
Flowchart diagram of enrolled patients.

**Figure 2:**
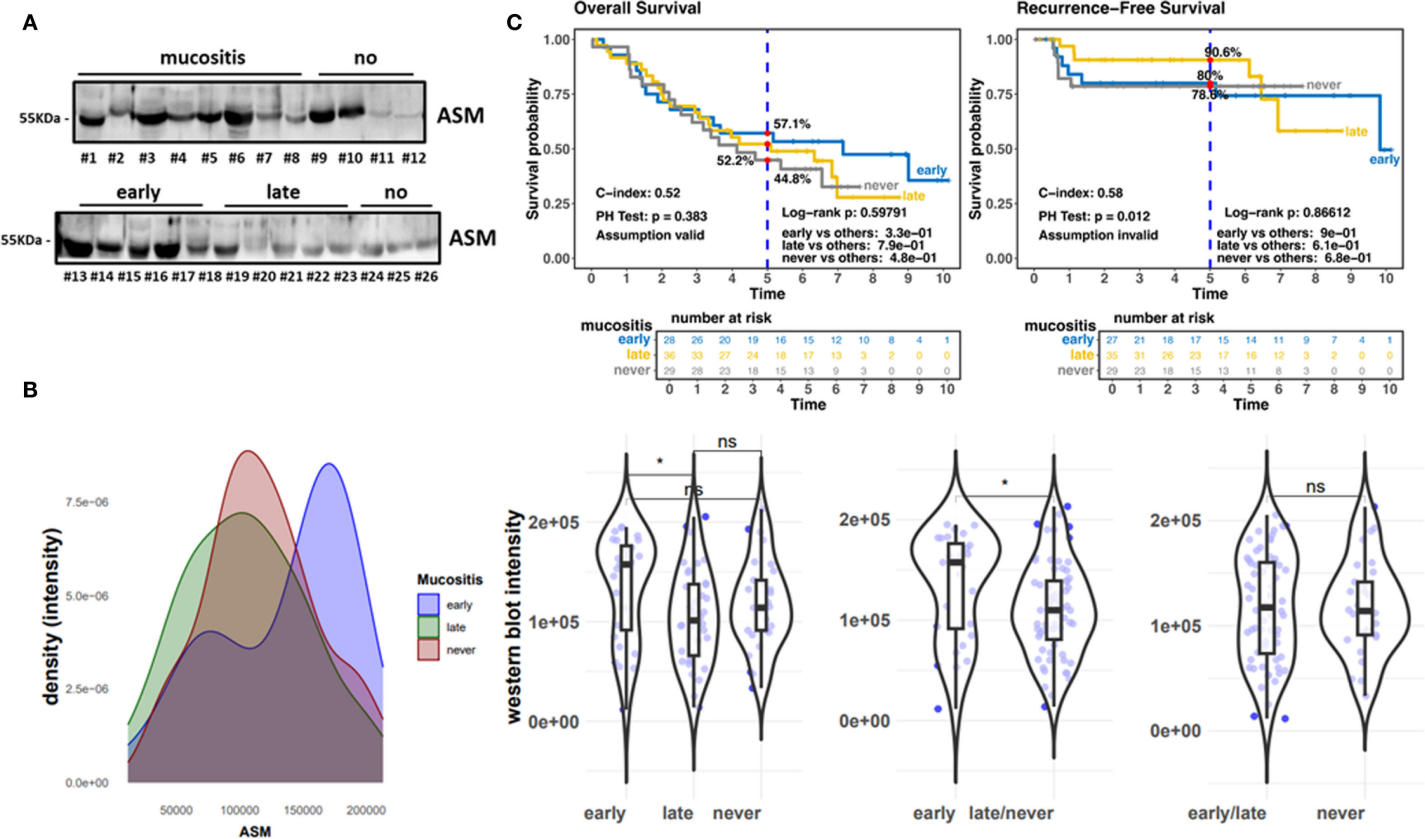
Historic cohort.**(A)** ASM expression levels in whole (unstimulated) saliva samples collected before the first RT application were analyzed by Western blot analysis. Equal protein amounts were loaded. Mucositis burden and molecular weight marker are indicated. Representative blots are shown. **(B)** Densitometrically quantified ASM signal intensities (arbitrary units) related to OM scores are shown. Individual dots represent individual patient samples (violin blots). **(C)** Overall survival (OS) and Recurrence-free survival curves for patients with early (n=42; blue line), late (n=32; yellow line) and no (n=35; grey line) mucositis. HR, hazard ratio and log-rank P are indicated. "*" significant; "ns"=not significant.

A total of 109 HNC patients were included in the historical cohort (**Figure 1**). The detailed patient and treatment characteristics are provided in **Table 1**. The most common sites of cancer were oropharynx (n=51, 46.8%), hypopharynx (n=33, 30.3%), and larynx (n=15, 13.8%). The major part of the participants had locally advanced disease: UICC stage IVA at 82 (75.2%) and IVB UICC stage at 10 (9.2%), and III at 11 (10.1%). 74 out of 109 patients developed OM (grade 3) during radiotherapy, of which 42 patients even displayed an early OM (grade 3) at a low radiation dose of < 34 Gy. Grade 3 OM developed in 50 (67.6%) patients after definitive RT and in 24 (32.4%) after adjuvant RT. 34 patients did not develop OM (grade 3) (**Table 1**, **Figure 2**). Thereby, the median RT dose in the group with grade 3 OM was 67.11 (59.4-70) Gy compared to 64.9 (57.3-70.3) in the group without severe OM. Unstimulated saliva was collected before the first radiotherapy application in a total of 109 HNC patients in this retrospective cohort. We screened ASM levels in respective samples via Western blot analysis (**Figure 2A**; **Supplementary Figure S1**). Densitometric quantification of respective signals revealed that ASM levels were increased in saliva samples of patients who developed mucositis early (**Figure 2B**). The distribution of ASM levels, indicated by the increased intensities in Western blot analysis, showed a significant increase in ASM in the saliva of patients who developed mucositis early. A corresponding analysis depending on the onset of mucositis (early, late, never) confirmed this assumption. Interestingly, the difference was significant when comparing the groups early mucositis (patients who had mucositis with < 34 Gy) versus late mucositis (patients who had grade 3 mucositis >= 34 Gy) and no mucositis. Overall and recurrence-free survival did not correlate with mucositis incidence (**Figure 2C**), even when survival of mucositis versus no mucositis was considered independently of the onset of mucositis (early/late) (**Supplementary Figure S2**).

**Table 1:** Phenotypic characterization of patients.

Mucositis grade 3	Prospective cohort	Historic cohort	Prospective cohort	Historic cohort
	No: n (%)		Yes: n (%)	
N	34 (43,6)/78 (100)	35 (32,1)/109 (100)	44 (56,4)/78 (100)	74 (67,9)/109 (100)
Age median	58,7	61,6	63,5	59,7
< 65 yrs	29 (85.3)	23 (65.7)	26 (59.1)	51 (68.9)
≥ 65 yrs	5 (14.7)	12 (34.3)	18 (40.9)	23 (31.1)
*Gender*				
female	4 (11.8)	5 (14.3)	11 (25.0)	18 (24.3)
male	30 (88.2)	30 (85.7)	33 (75.0)	56 (75.7)
Smoker	26 (76.5)	5 (14.3)	29 (65.9)	16 (21.6)
Non-smoker	8 (23.5)	2 (.7)	15 (34.1)	25 (33.8)
unknown		28 (80)		33 (44.6)
*Tumor site*				
CUP	2 (5.9)	0	1 (2.3)	0
Oral cavity	6 (17.6)	3 (8.6)	14 (31.8)	6 (8.1)
Oropharynx	14 (41.2)	14 (40.0)	20 (45.4)	37 (50.0)
Hypopharynx	12 (35.3)	13 (37.1)	8 (18.2)	20 (27.0)
Larynx	0	5 (14.3)	0	10 (13.5)
Nasopharynx	0	0	1 (2.3)	1 (1.4)
*UICC stage^1^*	*8. Edition*	*7.Edition*	*8. Edition*	*7.Edition*
I	2 (5.9)	0	8 (18.2)	1 (1.4)
I-II	0 (0.0)		1 (2.3)	
II	8 (23.5)	0	8 (18.2)	4 (5.4)
III	4 (11.8)	3 (8.6)	4 (9.1)	8 (10.8)
IVA	10 (29.4)	27 (77.1)	13 (29.5)	55 (74.3)
IVB	3 (8.8)	5 (14.3)	7 (15.9)	5 (6.7)
IVC	0	0		1 (1.4)
NA	7 (20.6)	0	3 (6.8)	0
*Tumor size*				
T1	4 (11.8)	5 (14.3)	2 (4.5)	8 (10.8)
T2	9 (26.5)	4 (11.4)	11 (25.0)	28 (37.9)
T3	11 (32.3)	8 (22.8)	15 (34.1)	19 (25.7)
T4	6 (17.6)	12 (34.3)	11 (25.0)	9 (12.2)
T4a	2 (5.9)	5 (14.3)	3 (6.8)	8 (10.8)
T4b	0 (0.0)	1 (2.9)	1 (2.3)	2 (2.7)
Tx	2 (5.9)		1 (2.3)	
*Nodal involvment*				
N0	7 (20.6)	3 (8.6)	3 (6.8)	9 (12.2)
N1	6 (17.7)	2 (5.7)	15 (34.1)	7 (9.4)
N2	4 (11.8)	10 (28.6)	5 (11.4)	9 (12.2)
N2a	1 (2.9)	0 (0.0)	1 (2.3)	6 (8.1)
N2b	5 (14.7)	10 (28.6)	6 (13.6)	17 (23.0)
N2c	6 (17.6)	9 (25.7)	8 (18.2)	22 (29.7)
N3	2 (5.9)	1 (2.8)	0 (0.0)	4 (5.4)
N3b	3 (8.8)		6 (13.6)	
*Distant Metastases*				
0	34 (97.1)	34 (97.1)	43 (98.6)	73 (98.6)
1	1 (2.9)	1 (2.9)	0	0
1a	0	0	1 (1.4)	1 (1.4)
*Grading*				
1	1 (2.9)	0	3 (6.8)	2 (2.7)
2	14 (41.2)	24 (68.6)	17 (38.6)	48 (64.9)
2-3	1 (2.9)		3 (6.9)	
3	13 (38.2)	11 (31.4)	17 (38.6)	22 (29.6)
4	1 (2.9)	0	0 (0.0)	1 (1.4)
NA	4 (11.8)	0	4 (9.1)	1 (1.4)
*HPV status*				
positive	15 (44.1)	NA	17 (38.6)	NA
negative	16 (47)	NA	17 (38.6)	NA
unknown	3 (8.9)		10 (22.8)	
*Mucositis grade 3 oncet^2^*				
r++	34 (100)	35 (100)	0	0
r	0	0	23 (52.3)	32 (43.2)
s	0	0	21 (47.7)	42 (56.8)
*Radiotherapy treatment*				
adjuvant	11 (32.4)	11 (31.4)	10 (22.8)	24 (32.4)
definitive	23 (67.6)	24 (68.6)	34 (77.2)	50 (67.6)
*RT dose (Gy) mean*	66.1 (37.8-70.4)	64.9 (57.3-70.3)	66.2 (58.8-70)	67.11 (59.4-70)

^1^It should be noted that tumor staging in the prospective cohort was performed according to the 8th edition of the UICC classification, whereas the historical cohort was staged using the 7th edition (28, 29). Differences in staging criteria - particularly concerning HPV-associated oropharyngeal carcinomas - should be taken into account when interpreting and comparing stage distributions across cohorts.

^2^The median dosis for grade 3 mucositis was 34 Gy (retrospective cohort) and 31.5Gy (prospective cohort). Patients without grade 3 mucositis were grouped as r++; patients who had grade 3 mucositis ≥ 34/31.5 Gy were grouped as r and ≤ 34/31.5 Gy were grouped as s.

**Figure 3:**
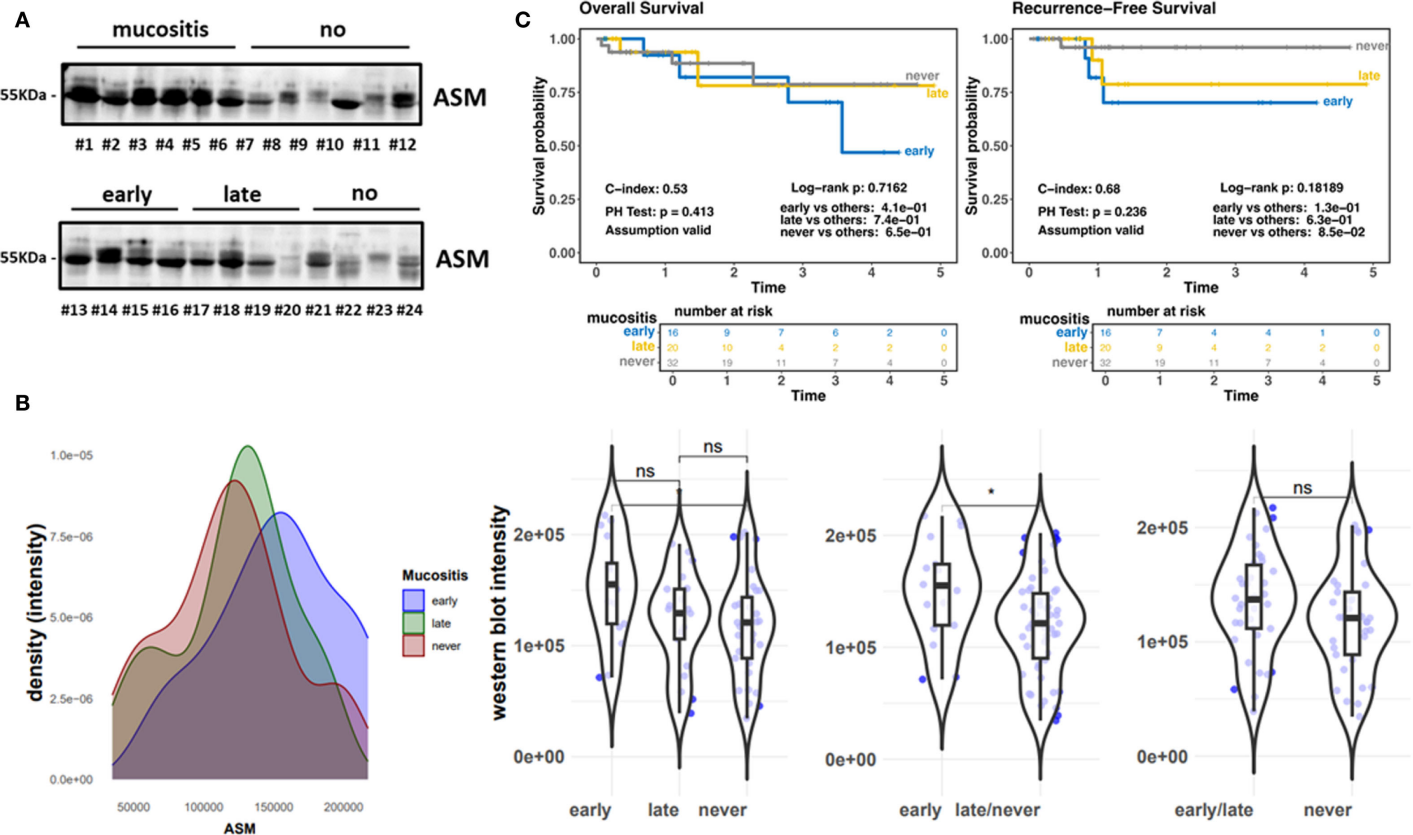
Prospective cohort (ZISStrans). **(A)** ASM expression levels in whole (unstimulated) saliva samples collected before the first RT application were analyzed by Western blot analysis. Equal protein amounts were loaded. Mucositis burden and molecular weight marker are indicated. Representative blots are shown. **(B)** Densitometrically quantified ASM signal intensities (arbitrary units) related to OM scores are shown. Individual dots represent individual patient samples (violin blots). **(C)** Overall survival (OS) and Recurrence free survival curves for patients with early (n=21; blue line), late (n=23; yellow line) and no (n=34; grey line) mucositis. HR, hazard ratio and log-rank P are indicated. "*" significant; "ns"=not significant.

**Figure 4:**
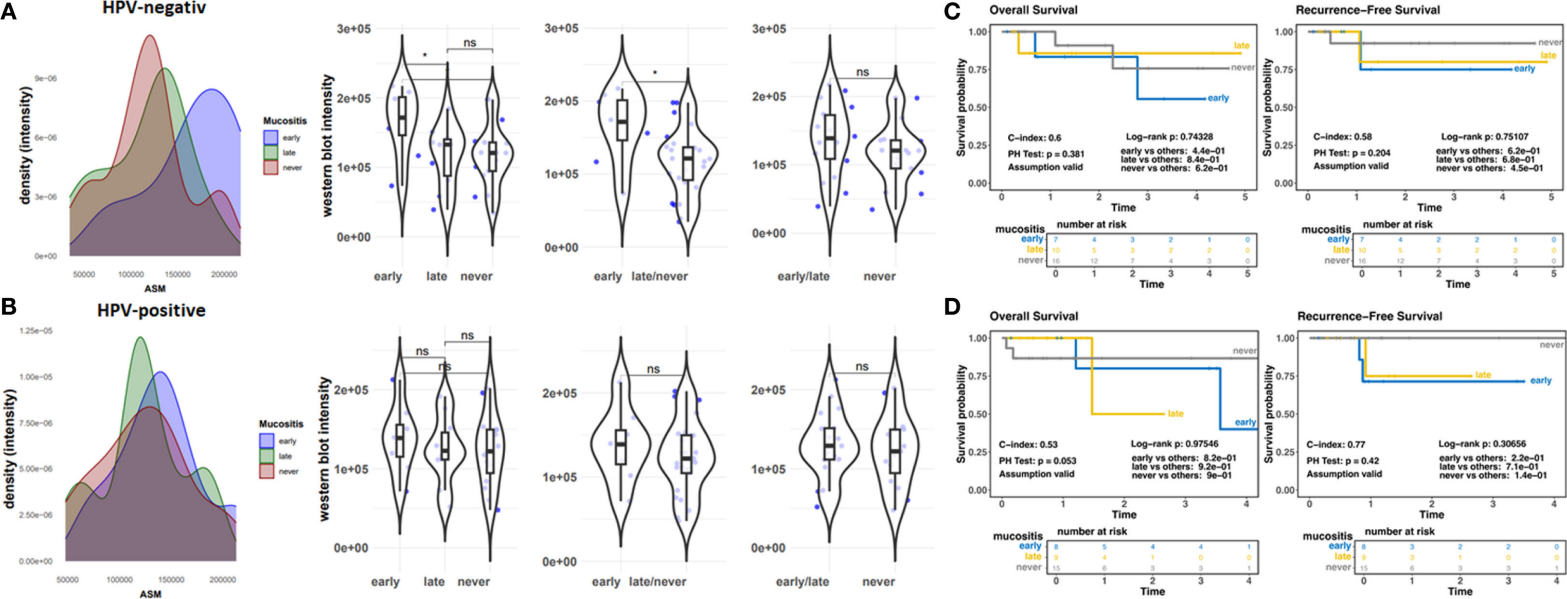
Prospective cohort (ZISStrans), HPV status **(A, B)** Densitometrically quantified ASM signal intensities (arbitrary units) as determined by Western blot analyses related to OM scores are shown for HPV-negative **(A)** and –positive **(B)** tumors. Individual dots represent individual patient samples (violin blots). **(C, D)** Overall survival (OS) and Recurrence free survival curves for HPV-negative **(C)** and-positive (D; n=40) samples. HR, hazard ratio and log-rank P are indicated. "*" significant; "ns"=not significant.

We investigated respective findings in a second cohort. A total of 78 HCN patients were included (**Figure 1**). Respective patient and treatment characteristics are listed in **Table 1**. The most common sites of cancer were the oropharynx (n=34, 43.6%), hypopharynx, and oral cavity each (n=20, 25.6%). The majority of participants had locally advanced disease: UICC IVA in 23 (29.5%) and IVB UICC stage in 10 (12.8%). HPV positivity was present in 37 (47.4%) patients. 44 out of 78 patients developed OM (grade 3) during RT, of which 21 patients even displayed an early OM (grade 3) at a low radiation dose of < 31.5 Gy. 34 patients did not develop OM (grade 3) (**Table 1**). The median RT dose in the group with grade 3 OM was 66.2 (58.8-70) vs. 66.1 (37.8-70.4) Gy without severe OM. Unstimulated saliva was collected here before the first RT application in a total of 76 HNC patients. No analysis could be carried out in 2 cases due to insufficient sample quantity. ASM levels were determined in respective samples via Western blot analysis (**Figure 3A**; **Supplementary Figure 1**) and confirmed elevated ASM levels in saliva patients who developed mucositis. The distribution of densitometrically quantified signals showed increased ASM levels in saliva samples of patients developing mucositis early and verified the finding from the historical cohort (**Figures 3B, C**). The corresponding analysis depending on the onset of mucositis confirmed the significant difference when comparing the groups early mucositis (patients who had mucositis with < 31.5 Gy) versus late mucositis (patients who had grade 3 mucositis >= 31.5 Gy) together with no mucositis. Overall and recurrence-free survival did not correlate with mucositis incidence (**Figure 3C**), even when survival of mucositis versus no mucositis was considered independent of the onset of mucositis (early/late) (**Supplementary Figure S2**), although patients developing mucositis might trend towards better overall- and recurrence-free survival. The distribution of densitometrically quantified ASM levels did not correlate with T stages or regional lymph node metastasis in both cohorts (**Supplementary Figure S3**). Considering HPV status, a parameter that was available in the second (prospective) cohort only, elevated ASM levels were found particularly in the saliva of HPV-negative patients who developed early mucositis, while no effect was observed in the HPV-positive samples (**Figure 4**). An additional determination of enzyme activity also showed a correlation with increased activity and the development of early onset of mucositis (**Supplementary Figure S4**). However, HPV status in combination with mucositis development had no influence on overall and recurrence-free survival (**Figure 4**; **Supplementary Figure S5**).

## 4 Discussion

A complex interplay of various risk factors, such as patient characteristics, tumor microenvironment, cancer treatment, and supportive care protocols, determines the risk of developing OM. However, based on individual variability in toxic mucosal responses, the ability to predict which patients are at risk of OM is almost impossible at present; but it might become possible. Our aim was to determine if ASM could be detected in the human saliva of HNC subjects and if this could be correlated with the degree and/or onset of OM. As a painful and debilitating acute oral disease, OM dramatically impairs quality of life and care, but can also disrupt cancer patients’ treatment plans due to repeated interruptions of radiotherapy, complete discontinuation, and dose adjustments. The presented results suggest, that increased ASM levels in saliva of patients prior (RT) treatment bear the potential to predict the onset of mucositis. This in turn could already have an impact on treatment schedules. Common screening methods of potentially predictive markers or predictive models altogether could be used to identify high-risk patients and thus support clinical decision-making, ultimately improving treatment planning for OM prevention and treatment. Based on dose-volume histogram parameter, radiomics, and dosiomics features, a normal tissue complication probability model was developed recently for the successful prediction of grade ≥ 2 acute oral mucositis in HNC patients undergoing (carbon-ion) radiation therapy (30). A recent publication also successfully demonstrated the performance of machine learning in predicting OM risk in patients who underwent radiotherapy to the head and neck region (31).

As an alternative biofluid in the diagnoses and prognosis of diseases, saliva represents a non-invasive, easy-collection oral biofluid for the analyses of medical conditions of an individual, for laboratory and clinical diagnosis, for planning approaches to prognosis and for patient monitoring and management as well (32, 33). In addition to its non-invasive features, richness in substances, and the huge amount make saliva testing a vital method for clinical applications (34). Several candidate proteins were identified (using mass spectrometry) in saliva samples from HNC patients that differ significantly between OM and non-OM groups (24). Generally, salivary cytokines and particularly inflammatory cytokines provide indicative information about oral conditions, with the cytokines IL-1β, IL-2, IL-6 and TNF-α correlating with the severity of oral mucosal tissue damage (35). Inflammatory mediators together with OM grade and oral mucosal dryness investigated in cancer patients and healthy volunteers confirmed that salivary IL-6, IL-10, and TNF levels could serve as biomarkers for OM occurrence and grade in patients with cancer (8). Likewise, elevated ASM levels in saliva of patients prior RT could serve as biomarkers for OM grade. Sphingomyelinases in general are key enzymes in sphingolipid metabolism that convert sphingomyelin to ceramide, thereby modulating membrane structures and finally triggering signal transduction involved in cell proliferation, apoptosis and differentiation caused by the extensive spatial lipid reorganization (36, 37). The involvement of the ASM/ceramide signaling pathway in the action of RT is already known (37–39), and participation in OM is also accepted. OM generally results from a series of dynamic and interactive molecular and cellular events, which can be roughly divided into 5 phases, the initiation, primary damage response, signal amplification, ulceration and healing stages (13). Although all elements of the mucosa are involved, RT-induced endothelial apoptosis might be a potential initiator of mucositis, which turned out to be ASM/ceramide-dependent (40). Although it is not clear what causes the per se increased ASM levels in the saliva of HNC patients that could be detected before therapy, possibly the tumor burden itself, an altered oral flora or already other (therapy-) induced inflammation, these levels can adversely promote OM events in the phase of signal amplification by amplifying inflammation and pro-apoptotic signals. Although not yet investigated in HNC, levels of ceramide were shown to be significantly decreased in certain cancers, which suggested that in at least some cancers this is based on down-regulated ASM levels that might, at least partially, account for reduced apoptosis responses here directing the cells more toward proliferation (41). Several studies already suggested that ASMase plays a role in the pathophysiology of common diseases, particularly systemic inflammation and sepsis, as increased ASM levels were detected here e.g., in plasma of septic patients compared with healthy control subjects (41, 42). In addition, preclinical evidence arises that genetic deficiency or pharmacologic inhibition of ASM overcomes inflammation-induced organ failure and improves survival particularly at the level of the vasculature (43–45). Thus, functional ASM inhibitors might be considered as a pharmacological treatment strategy to favor OM outcome. The ASM-ceramide system contributes to numerous diseases, among other things, through inflammasome activation (46). The best-studied inflammasome complex is the NOD-like receptor 3 (NLRP3), which activation and proinflammatory cytokine production (via Nrf2 signaling) are particularly involved in oral diseases (47). ASM in turn, and particularly ceramide associated membrane raft signaling platforms, were already shown to contribute to the activation of NLRP3 inflammasomes and thus NF-κB signaling mediating inflammatory responses (48–50). Thus, beside considering ASM as promising clinical biomarker marker, ASM as a potential therapeutic target in OM that warrants further investigations. Today, OM treatment focuses on symptom relief, especially in the early stages of this adverse event. The identification of new potential candidate molecules would pave the way for further research into new diagnostic, preventive, and therapeutic targets against this tissue damage. Within that scenario, it was for example shown that IL-17RA plays an important protective role in radiation-induced OM by limiting excessive inflammation during the ulcerative phase. Its absence leads to increased infiltration of immune cells, epithelial apoptosis and impaired regeneration, resulting in severe mucosal damage. These results highlight the importance of IL-17RA for maintaining mucosal integrity and point to potential therapeutic implications for targeting the Th17 pathway in cancer treatment. Currently, there is very limited consistent evidence regarding the role and function of protective markers, especially in radiation-induced OM (51). Oral mucosal barrier protectants, including agents protecting mucosal integrity and reducing inflammation, turned out to be important for oral mucosal barrier immunity. The Th17 cellular response may be a critical factor in inflammatory diseases of the oral mucosa with “pathogenic Th17”, an important subset of CD4+ T cells that mediate dementrial tissue effects, comprising a potential therapeutic target for treating oral mucosal inflammatory disorders as use of Anti-IL-17A monoclonal antibodies or inhibition of the Th17/Tc17 axis alleviated diseased states (52–54).

Cervical lymph node metastasis (LNM) is one of the most important factors for determination of appropriate treatment and thus one of the most important parameters determining prognosis in patients with HNSCC, with the presence of only one positive lymph node being associated with a decrease survival by up to 50% in most HNC (55, 56). The level of LNM is even an independent prognostic factor for survival in patients with locally advanced HNC especially for patients with oral cavity, oropharynx and larynx HNC (57, 58), and LNM numbers were even associated with a higher risk of distant metastases (59). Likewise, stage and HPV status are now recognized as major determinants of HNC prognosis, at least in Western regions (58, 60, 61), with T classes 1–2 showing improved survival compared to T classes 3–4 (58). We could not identify any potential correlation between ASM levels and TNM stages in our cohorts; and overall as well as recurrence-free survival did not correlate with mucositis incidence, which is in line with previous findings that revealed no long-term impact of severe acute grade 3 OM on oncological endpoints (25).

Conclusively, the determination of ASM content has the potential for (early) detection of high-risk candidates for (early) mucositis prior treatment start. These results were collected in one cohort and confirmed in a second independent cohort; however, limitations must be noted. The recruited patients were treated at only one facility, which may not be sufficiently representative for the overall population. Another point is that the saliva samples, especially those from the first cohort, were frozen for a longer period and were not analyzed fresh immediately after collection. This could have resulted in artifacts of cellular debris, which, strictly speaking, could also be present in every sample. The saliva volumes from the first cohort, on the other hand, were insufficient in terms of volume to allow for additional activity measurements. A total ASM activity measurement would make a potential screening procedure as an additional indicator for early mucositis even easier than determining levels via Western blot analysis, as this method is not only faster but also does not depend on the quality of a suitable ASM antibody. We further recommend considering the collection of salivary samples prior to the initiation of radiotherapy in order to identify particularly vulnerable patients at an early stage. Such early detection may allow for intensified monitoring and supportive measures during treatment, potentially reducing the risk of therapy interruptions or premature discontinuation.

## Data Availability

The original contributions presented in the study are included in the article/**Supplementary Material**. Further inquiries can be directed to the corresponding author.
