# Supplementary material for: Increased acid sphingomyelinase levels in saliva as oral mucositis severity predictors
Source: Front Oncol. 2025 Sep 25;15:1613884. doi: 10.3389/fonc.2025.1613884 (PMC12507555; doi:10.3389/fonc.2025.1613884)
Supplement: Supplementary file 1 [file DataSheet1.pdf]

Supplementary Material

1 Supplementary Figures

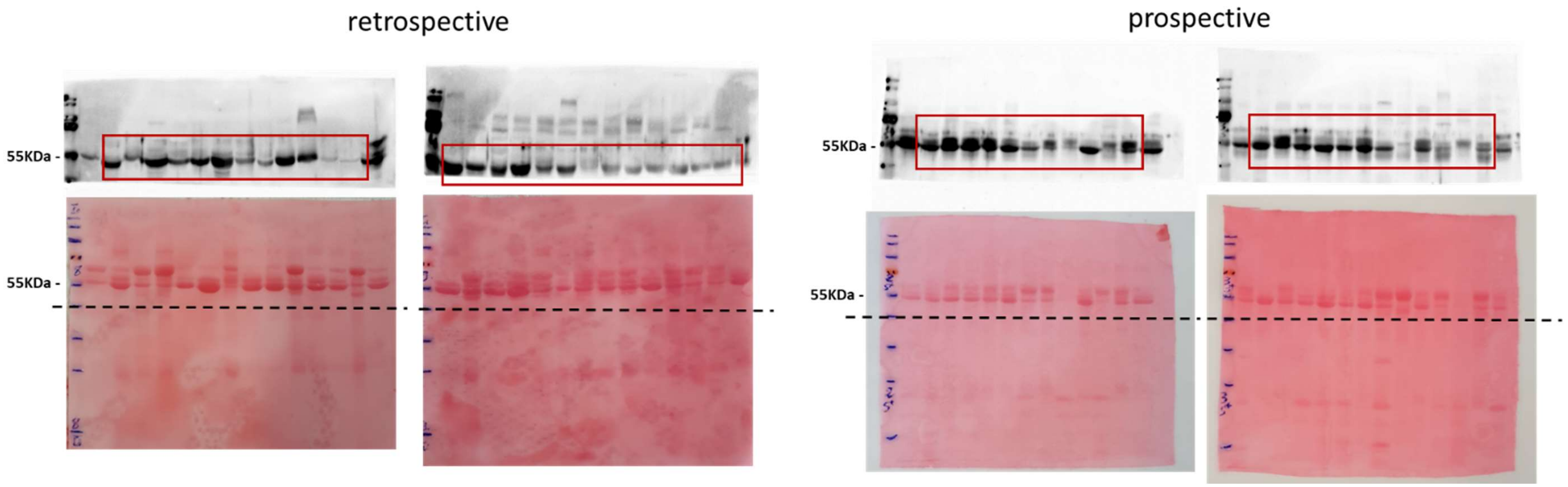

Supplementary Figure S1

Full gels of cropped gels (emphasized by a red rectangle) as shown in Figure 1A (right) and 2A (left). Equal protein amounts of total saliva protein estimates were loaded for SDS-PAGE gel electrophoresis. Blotted proteins were visualized on PVDF membranes using Ponceau S Staining (lower panels). Membranes were cut at around the 40 kDa marker (indicated by dashed line) and the upper membrane parts (upper panels) were washed prior primary antibody incubation (with goat anti ASM antibody; overnight at 4°C). Horseradish peroxidase-conjugated (anti goat) secondary antibody was used for chemiluminescent detection. (Lower membrane parts were used for other epitope investigations; not shown).

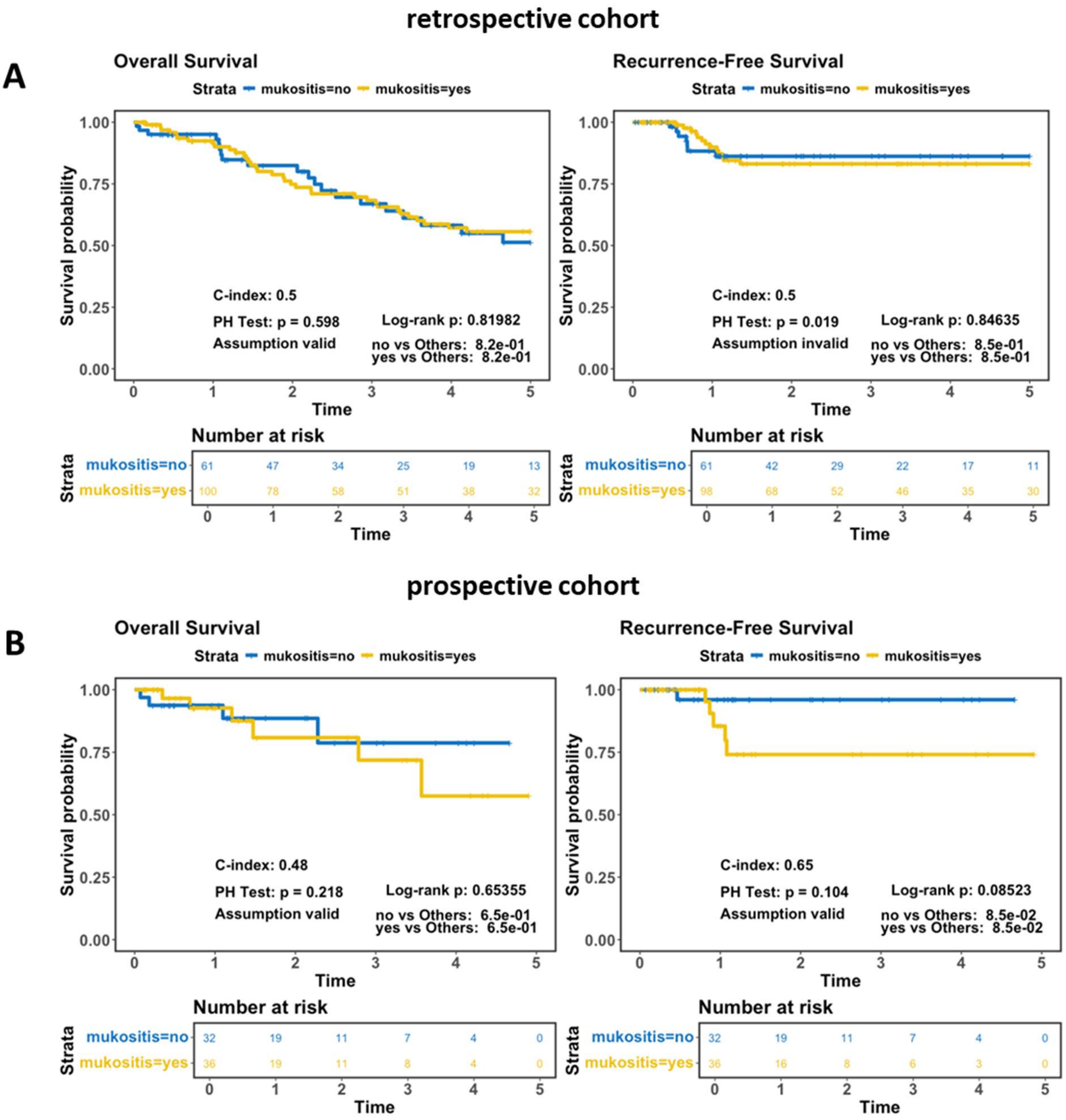

Supplementary Figure S2

Overall survival (OS) and regression-free survival curves for patients with (early and late) and without mucositis. HR, hazard ratio and log-rank P are indicated.

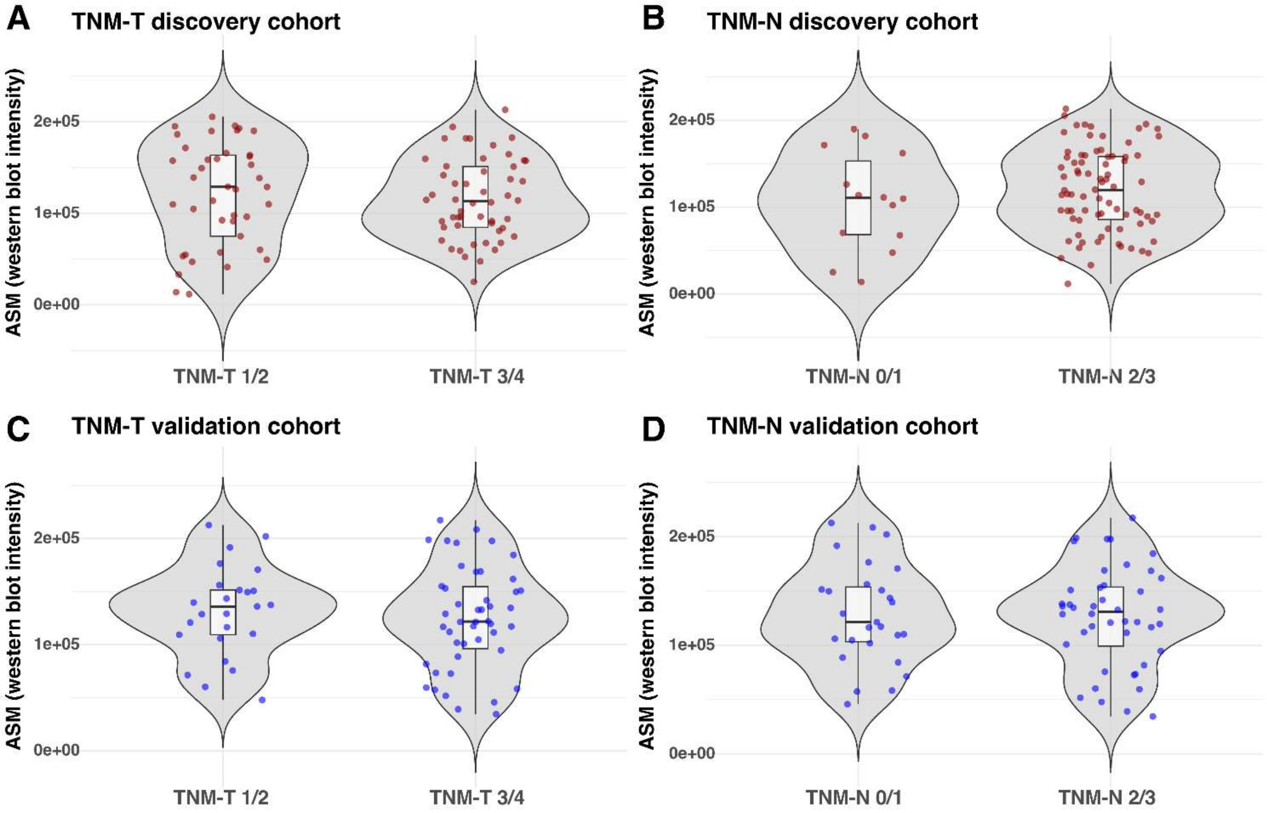

Supplementary Figure S3

Densitometrically quantified ASM signal intensities (arbitrary units) related to early versus advanced TMN classification: T staging/ staging multiple sites of malignancy (TMN-T) for the historic (discovery) cohort (A) and the prospective (validation) cohort (C), and respective relations for N staging/ regional lymph node metastasis (TMN-N) for both cohorts (B, D).

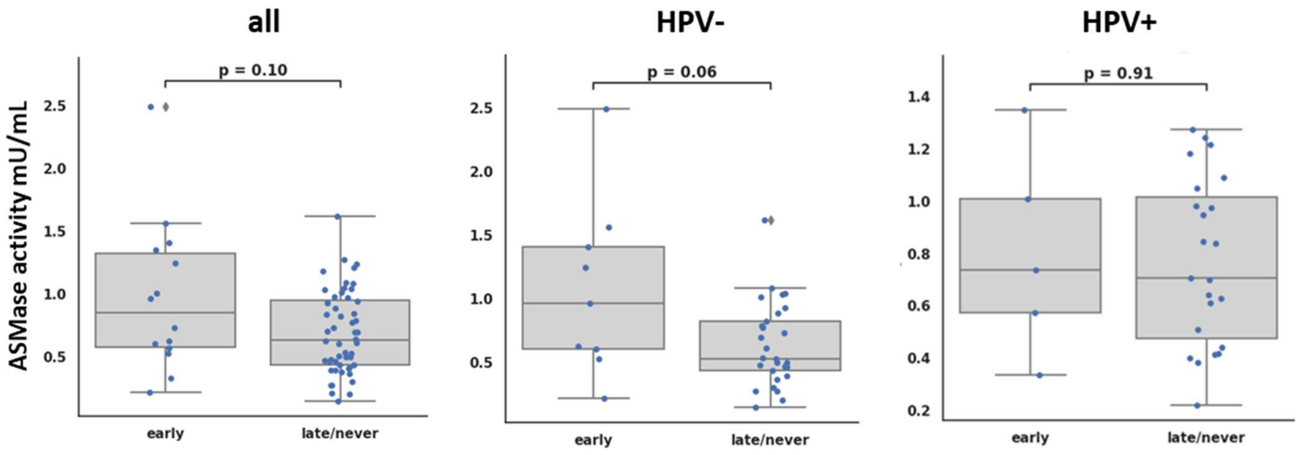

Supplementary Figure S4

ASMase activity (mU/mL) in unstimulated saliva samples (prior RT) was determined following conversion of sphingomyelin to phosphorylcholine and ceramide at pH 5.0. Activity was calculated using a choline standard curve. Individual dots represent individual patient samples measured in duplicates each.

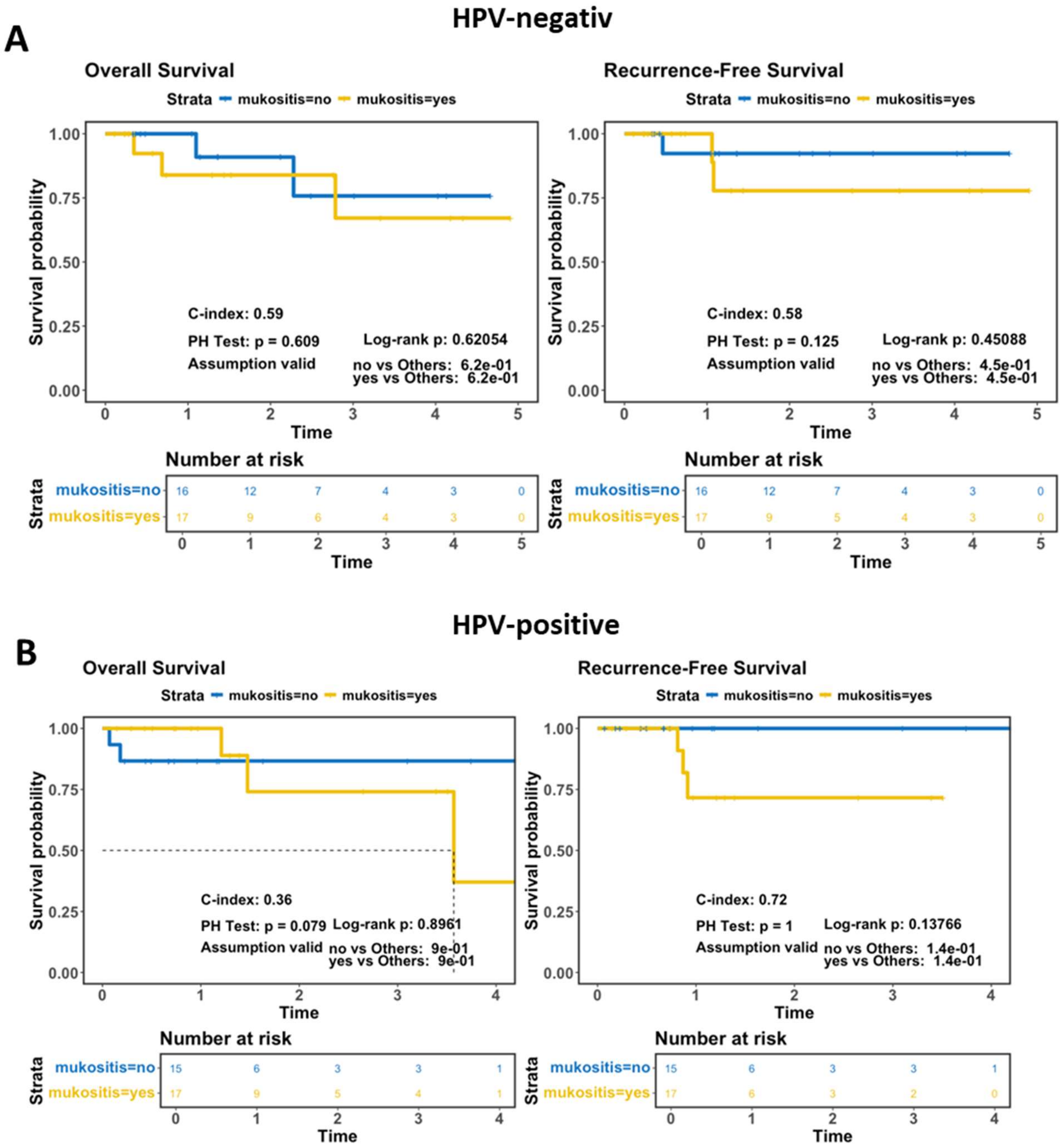

Supplementary Figure S5

Overall survival (OS) and regression-free survival curves for patients with (early and late) and without mucositis and according to the HPV status. HR, hazard ratio and log-rank P are indicated.
